# Research progress on the mechanisms related to myocardial injury in rats with myocardial ischemia-reperfusion injury treated by acupuncture at neiguan (PC6) acupoint: A narrative review

**DOI:** 10.1097/MD.0000000000044698

**Published:** 2025-12-12

**Authors:** Qiuping Xin, Zhixu Gao, Jianen Guo, Yuman Wang, Zhibin Zhang, Yan Liu, Bingran Yang, Jiaying Sun

**Affiliations:** a Department of General Medicine, Affiliated Hospital of Chengde Medical University, Chengde, Hebei, China; b College of Traditional Chinese Medicine, Chengde Medical University, Chengde, Hebei, China.

**Keywords:** acupuncture, mechanism, myocardial ischemia-reperfusion injury, Neiguan point

## Abstract

Acute myocardial infarction presents a considerable clinical burden with high morbidity and mortality rates, with reperfusion therapy being the primary treatment modality. However, following revascularization myocardial ischemia-reperfusion injury can trigger secondary functional cardiac damage, accelerating necrosis of cardiomyocytes. This poses a significant challenge to patient health and well-being. Acupuncture therapy, a cornerstone of traditional Chinese medicine, holds promise in addressing cardiovascular ailments. Neiguan point is an 8 meridians meeting point, belonging to the hand jueyin pericardium meridian, and connected with yinwei meridian. It has the effects of calming the heart and calming the spirit, regulating qi and relieving pain. Modern medical research has confirmed that it can regulate cardiac function through the neuro-humoral-immune network, protect myocardial function, and have a good effect in the treatment of cardiopulmonary diseases. When strategically combined with other acupuncture points, the Neiguan stimulation point exhibits potential in mitigating myocardial ischemia-reperfusion injury. This study explores the impact of acupuncture at the Neiguan point in rats with myocardial ischemia-reperfusion injury, aiming to shed light on preventive and therapeutic strategies for this condition.

## 1. Introduction

Ischemic cardiomyopathy, frequently triggered by acute ischemia and hypoxia of the coronary arteries has a high clinical incidence. It is prevalent among middle-aged and elderly individuals, posing significant threat to safety of the patient.^[1]^ When patients with ischemic cardiomyopathy undergo reperfusion therapy it can exacerbate tissue damage leading to ischemia-reperfusion injury. This process upregulates oxygen radical expression, triggering inflammatory reactions and inflicting substantial harm on cardiomyocytes.^[2,3]^ Acupuncture therapy characterized by its simplicity, minimal side effects, and requirement for multiple clinical sessions, offers a promising avenue for intervention. Acupuncture has the potential to mitigate myocardial tissue damage by modulating the body’s stress response, thus facilitating the activation of intrinsic adaptive mechanisms that can reduce the severity of such damage.^[4]^ The Neiguan point demonstrates enhanced therapeutic efficacy in managing cardiothoracic diseases and holds promise in treating ischemia-reperfusion injury. “Ling Shu · Jing Mai” records that it “causes heart pain,” and modern clinical practice has also confirmed its definite curative effect on coronary heart disease and angina pectoris. Acupuncture targeting this point can help sustain the internal equilibrium of the body and mitigate associated pathological damage.^[5]^ This study serves as a narrative review evaluating the efficacy of acupuncture at Neiguan acupoint in treating myocardial ischemia-reperfusion injury. The literature search was conducted across databases including PubMed, Web of Science, China National Knowledge Infrastructure, and Wanfang, covering the period from database establishment to December 2023. Search terms encompassed “acupuncture,” “Neiguan acupoint,” “myocardial ischemia-reperfusion injury,” “rats,” “pathogenesis,” along with corresponding English equivalents, utilizing both subject-specific keywords and free-text searches.

Among the studies included in this review (see Table 1), the experimental rats primarily involved Sprague Dawley rats and Wistar rats. Most studies utilized male rats, with only 2 studies employing female rats. There were certain differences in the selection of rat strains across the studies, parazacco spilurus subsp. spilurus, which may be related to the breeding traditions of different laboratories. The differences in gender selection, parazacco spilurus subsp. spilurus, might influence the detection results of inflammatory factor levels and the degree of myocardial injury.

**Table 1:** Key mechanisms and related research results of acupuncture at Neiguan point in improving myocardial ischemia-reperfusion injury in rats.

Mechanism of action	Key details	Finding
Inhibit oxidative stress	Increased SOD activity and Nrf2 expression, decreased LDH and MDA levels	Acupuncture at guan yin acupoint can enhance the activity of antioxidant enzymes, reduce oxidative products and reduce myocardial oxidative injury
Regulate mitochondrial function and energy metabolism	Improve mitochondrial membrane potential, reduce cytochrome C release, and maintain ATP synthesis	Acupuncture can repair mitochondrial structure, regulate energy metabolism and protect myocardial function
Reduce inflammation damage	Reduce TNF-α, IL-6 and other inflammatory factors, reduce leukocyte aggregation, regulate vascular active substances	Acupuncture at guan yin point can inhibit the activation of inflammatory pathway, protect microvessels and reduce inflammatory infiltration
Improve endothelial function	Activate Akt/eNOS pathway and regulate CGRP, NO and other vasoactive substances	Electroacupuncture stimulation can improve endothelial secretion function and maintain vascular contraction and relaxation balance
Reduce calcium overload	Enhance the activity of Ca^2+^-ATPase and Na^+^–K^+^-ATPase to maintain calcium homeostasis	Electroacupuncture pretreatment can improve calcium imbalance and reduce myocardial contractile dysfunction
Reduce apoptosis	Reduced caspase-3 activity, inhibited cardiomyocyte apoptosis, and reduced CK-MB level	Acupuncture can reduce the infarct area and inhibit the process of autophagy and apoptosis
Regulate the central nervous system	Reduced the expression of 5-HT and β3-ADR, and increased the concentration of DA and NE in hypothalamus	Regulate the level of neurotransmitters, maintain the balance of cardiovascular activity, and reduce stress damage
Increase protective proteins	The expression of heat shock proteins such as HSP70 and HSP27 was increased	The expression of protective proteins increased at different phases after acupuncture, which enhanced the stress adaptability of cells
Regulate related signaling pathways	Regulate miRNA-214/Ca^2+^ pathway, MAPK pathway and vagus nerve-mast cell pathway	Stimulation of Neiguan acupoint can reduce cell death and protect myocardium through multiple signal pathways

5-HT = 5-hydroxytryptamine, ATP = adenosine triphosphate, CGRP = calcitonin gene related peptide, CK-MB = creatine kinase-MB, DA = dopamine, HSP27 = heat shock protein 27, HSP70 = heat shock protein 70, IL-6 = interleukin-6, LDH = lactate dehydrogenase, MAPK = mitogen-activated protein kinase, MDA = malondialdehyde, miRNA-214 = microRNA-214, NE = norepinephrine, NO = nitric oxide, Nrf2 = NF-E2-related factor 2, SOD = superoxide dismutase, TNF-α = tumor necrosis factor-α, β3-ADR = recombinant adrenergic receptor beta3.

## 2. Inhibition of oxidative stress

Oxidative stress plays a crucial role in various physiological processes such as immunity, body metabolism, growth, and development, in healthy organisms. A moderate stimulation of stress response can prompt the elimination of abnormally expressed cells, ensuring the maintenance of the internal equilibrium of the body. However, in the context of ischemia-reperfusion injury there is a sustained release of oxygen free radicals, leading to oxidation, antioxidant imbalance, and oxidative damage.^[6]^ The excessive release of oxygen free radicals can detrimentally affect unsaturated fatty acids within membrane components leading to an increased and continuous secretion of lipid peroxidation products. This process can inflict damage upon membrane structures within the endoplasmic reticulum and mitochondria, ultimately leading to myocardial ischemia-reperfusion injury.^[7]^ Clinical pre-research studies have shown that acupuncture has strong antioxidant and reactive oxygen capacity, which can continuously increase the activity of oxygen free radical scavenging enzymes, reduce the expression of lactate dehydrogenase and malondialdehyde, increase the level of superoxide dismutase (SOD), and inhibit the oxidative damage of myocardial cells.^[8,9]^ Studies indicate that acupuncture exhibits a robust antioxidant capacity effectively reducing levels of reactive oxygen species. Consequently, this leads to a sustained increase in the activity of oxygen free radical scavenging enzymes, a reduction in the expression of lactate dehydrogenase and malondialdehyde, an elevation in the level of SOD, and inhibition of oxidative damage to cardiomyocytes. Stimulation of the Neiguan point has been observed to prompt a continuous increase in SOD activity, upregulate the expression of NF-E2-related factor 2, and reduce gene damage, resulting in a sustained elevation in the expression of downstream antioxidant enzymes. This process facilitates the scavenging of oxygen free radicals and inhibits damage to cardiomyocytes within the organism.^[10,11]^

## 3. Regulation of mitochondrial function and energy metabolism

Mitochondria play a critical role in cellular metabolism by converting nutrients into adenosine triphosphate (ATP) through enzyme catalysis, which is essential for normal cellular activities. However, ischemia and hypoxia can disrupt electron transfer in the mitochondria leading to anaerobic glycolysis and a reduced expression of ATP.^[12]^ With the increasing of clinical hypoxia time, it can lead to cellular acidosis, which can aggravate the impairment of bioenzyme activity, aggravate the condensation of nuclear chromatin, and continuously affect the function of ATP synthase, which can lead to continuous impairment of ATP synthesis and reduce energy supply.^[13]^ During the reperfusion injury process in organisms rapid changes in ion flux can occur, correcting cellular acidosis. The swift normalization of ion flux can lead to rapid alterations in mitochondrial membrane permeability causing the disappearance of the mitochondrial membrane potential. Consequently, abnormal mitochondrial structural function ensues, accompanied by the release of cytochrome C, which damages the mitochondrial structure and reduces respiratory energy production efficiency.^[14]^

Animal studies demonstrate that acupuncture therapy can regulate mitochondrial function, influence the body’s energy metabolism, protect myocardial ischemia-reperfusion injury, repair mitochondrial damage, and safeguard mitochondrial function and structure.^[15,16]^ Research indicates that acupuncture treatment can protect cardiac function in rats with ischemia-reperfusion injury, enabling cells to degrade damaged mitochondria through lysosomal enzymes and digesting cytoplasmic and mitochondrial autophagosomes within the double-membrane structure. Clinical studies show^[17]^ that maintaining mitochondrial stability through acupuncture at Neiguan acupoint can reduce autophagocytes in ischemic myocardial tissue, inhibit mitochondrial-ligand binding to lysosomes, alleviate myocardial ischemia-reperfusion injury, and maintain a balance between cellular autophagy and apoptosis, thereby protecting rat myocardium.

## 4. Reduce inflammatory damage

The occurrence of myocardial ischemia-reperfusion injury can impact the inflammatory response of the body by inducing an increase in leukocyte count, leading to sustained infiltration and subsequent damage to the myocardial tissue. This process triggers the activation of relevant neutrophils, perpetuates systemic stimulation of the body, enhances the expression of inflammatory factors, and damages vascular endothelial cells.^[18]^ Acupuncture at the Neiguan point can exert a potent inhibitory effect on the secretion of chemokines within the body, diminishing the accumulation of leukocytes and reducing the extent of microvascular damage.

### 4.1. Reducing leukocyte aggregation

During myocardial ischemia and reperfusion the secretion of tumor necrosis factor-α, vascular cell adhesion molecule-1, interleukin-8, and interleukin-6 increases, leading to enhanced leukocyte adhesion to the vascular endothelium. This process, triggers inflammatory responses and leukocyte-related effects, ultimately causing damage to cardiomyocytes and resulting in microcirculation disorders.^[19]^ Studies indicate that acupuncture on the Neiguan point can inhibit the inflammatory response in the organism^[20]^ leading to a reduction in the expression level of inflammatory factors. Moreover, electroacupuncture at the Neiguan point has been shown to decrease the expression of high mobility group protein B1, inhibit the infiltration of inflammatory cells in the myocardial tissues, and lower the expression levels of tumor necrosis factor-α and interleukin-6, And may inhibit the activation of mitogen-activated protein kinase (MAPK) signaling pathway, promote nuclear factor-k-gene binding (NF-kB) nuclear translocation and NF-kB nuclear translocation plays a role (NF-kB can promote the activation of inflammatory cytokines). Additionally, it can activate the MAPK signaling pathway, facilitating the nuclear translocation of NF-kB, which subsequently activates inflammatory cytokines. Thus, suppressing high mobility group protein B1 expression can mitigate the inflammatory response, indicating a correlation between the acupuncture on the Neiguan point and the modulation of the inflammatory pathway.

### 4.2. Protection of microvessels

Animal experiments have revealed that myocardial ischemia-reperfusion injury in rats can elevate the levels of endothelin and thromboxane B2, leading to vasoconstriction and platelet aggregation in blood vessels, ultimately resulting in a continuous reduction in the expression level of 6-ketoprostaglandin F1α.^[21]^ Acupuncture at the Neiguan point has been shown to regulate vasoactive substances, increase nitric oxide expression, and promote the dilation of the blood vessels. High-frequency electroacupuncture stimulation at the Neiguan point demonstrates a more pronounced effect on nitric oxide elevation, as well as enhancing the vasodilator effect of calcitonin gene related peptide (CGRP).

In clinical practice, during reperfusion, there may be excessive vasodilation of the blood vessels, exacerbating myocardial reperfusion injury. However, utilizing electroacupuncture to stimulate the Neiguan point can help maintain a certain level of CGRP post reperfusion, thus effectively dilating blood vessels to a specific concentration. This approach aids in protecting cardiomyocytes of the patient and reducing reperfusion injury.

## 5. Improvement of endothelial function

The occurrence of ischemia and hypoxia in cardiomyocytes can lead to endothelial damage in coronary arteries, triggering the secretion of adhesion molecules and an increase in intravascular reactive oxygen species, ultimately causing cardiomyocyte injury. The elevated levels of reactive oxygen species can disrupt the secretion of vasoconstrictive and vasodilatory factors, diminishing blood flow rates and causing myocardial damage.^[22]^ Research demonstrates that applying warm acupuncture at different phases to ischemia-reperfusion injury rats enhances vasomodulatory substance expression, reduces infarcted tissue size, and protects ischemic myocardium. Electrical acupuncture stimulation at Neiguan acupoint improves endothelial cell secretion function and mitigates ischemia-reperfusion injury, with mechanisms potentially involving enhanced neural-muscular connectivity between the acupoint and myocardium, as well as improved cardiac function.^[23]^ Studies indicate that stimulating ischemia-reperfusion-injured rats with warm acupuncture at different time phases can upregulate the expression of vasoactive substances and decrease the infarct area, thereby protecting the ischemic myocardium in rats. Electroacupuncture stimulation of the Neiguan point has been observed to enhance the secretion function of endothelial cells, reduce ischemia-reperfusion injury, strengthen the connection between the Neiguan point and the myocardium, and enhance the cardiac function of the body.^[24,25]^ Furthermore, studies have demonstrated that acupuncture at the Neiguan point increases the expression of vasoactive substances and activates the Akt/eNOS pathway. Ischemia-reperfusion injury induces a stress response, increasing the concentration of CGRP and dilating blood vessels, which can exacerbate myocardial reperfusion injury. Electroacupuncture treatment at the Neiguan point can reduce the expression of CGRP, alleviate the stress response, and protect ischemic myocardium.

## 6. Reduction of calcium overload

The dynamic balance of calcium ions (Ca^2+^) plays a pivotal role in cardiomyocytes, significantly impacting the contractile function of the heart. Insufficient ATP supply can lead to dysfunction of the sodium–potassium (Na^+^–K^+^) pump and Ca^2+^ pump, resulting in the increased accumulation of Na^+^ and Ca^2+^ within the cell. This accumulation can lead to calcium overload in cardiomyocytes, causing cardiac contractile dysfunction, ventricular reconstruction, and exacerbation of cardiomyocyte damage.^[26]^ Studies have shown that pretreatment of diabetic rats with electroacupuncture improves calcium homeostasis in myocardial ischemia-reperfusion injury.^[27]^ This intervention enhances the activities of Ca^2+^-ATPase, Mg^2+^-ATPase, and Na^+^–K^+^-ATPase. These findings suggest that pretreatment at the Neiguan point helps maintains calcium homeostasis in cardiomyocytes of rats, enhances the capacity to scavenge oxygen free radicals, reduces cardiac inflammatory reactions, and mitigates cardiac function injury.

## 7. Reduction of apoptosis

In cellular life processes, apoptosis is a complex and highly regulated mechanism that plays a significant role in myocardial ischemia-reperfusion injury. Studies have highlighted apoptosis as a central aspect of the injury mechanism, commencing during the early stages of myocardial ischemia and persisting throughout the ischemia-reperfusion injury.^[28]^ Apoptosis is influenced by a variety of apoptosis-regulating proteins, categorized into pro-apoptotic and anti-apoptotic proteins. Disruption of the equilibrium of these proteins under stress conditions can trigger cell death. The cysteinyl aspartate-specific proteinase family, particularly cysteine protease-3, plays a crucial role in regulating the apoptotic process.^[29]^

During myocardial ischemia, pretreatment with acupuncture has been shown to inhibit the apoptosis of cardiomyocytes and mitigate cardiac function injury.^[30]^ Additionally, acupuncture pretreatment in rats with myocardial ischemia-reperfusion injury improves cardiac function, reduces the expression of inflammatory factors in the organism, and decrease the area of their myocardial infarction.^[31]^ Acupuncture administered at 30 minutes of reperfusion demonstrates enhanced therapeutic effects, inhibiting both cell autophagy and apoptosis, while reducing the infarct area. Studies have indicated that acupuncture at the Neiguan point can inhibit pathological changes in rat myocardial tissue, decrease the level of Creatine Kinase-MB, and inhibit cardiomyocyte apoptosis to protect the myocardium.^[32,33]^

## 8. Regulation of the central nervous system

5-Hydroxytryptamine and opioid peptides are neurotransmitters located in the ventral-lateral area of the head part of the medulla oblongata. When activated, they play a role in inhibiting excessive tension in sympathetic nerves, preventing the generation of harmful stimuli, enhancing the stress tolerance of the heart, and protecting normal cardiomyocytes in the body.^[34]^ Experimental studies have demonstrated that acupuncture at the Neiguan point can decrease the expression of 5-hydroxytryptamine and mitigate reperfusion injury. This process aids in regulating the body’s nerve center and maintaining cardiovascular activity balance.^[35]^

Additionally, myocardial ischemia and hypoxia can increase the expression levels of the recombinant adrenergic receptor beta3, leading to the progressive decline of cardiac function.^[36]^ Research has shown that acupuncture at the Neiguan point can decrease recombinant adrenergic receptor beta3 expression, suppress transmission of injurious neural signals, and protect cardiomyocytes.^[37]^ The paraventricular nucleus of the hypothalamus plays a crucial role in vascular activity and can confer neuroprotective effects. In the current study, rats with myocardial ischemia-reperfusion injury underwent acupuncture at the Neiguan point resulting in down-regulation of neural electrical activity in the dorsal root of the spinal cord, elevation of dopamine and norepinephrine concentration in the hypothalamus and enhancement of the anti-neuronal mechanism.

## 9. Increase protective proteins

Cells increase the expression of protective proteins following stress stimulation. This upregulation particularly of heat shock proteins serves to trigger apoptosis, reduce cellular dysfunction, maintain internal environment stability, and prevent ischemia-reperfusion injury.^[38]^ Studies have demonstrated that acupuncture treatment at the Neiguan point for ischemia-reperfusion injury can augment the expression of heat shock protein 70.^[39]^ The results reveal a significant increase in heat shock protein 70 expression 48 hours post-acupuncture. Additionally, there is an elevation in heat shock protein 27 expression 24 to 48 hours following acupuncture treatment. These findings suggest that the protective effects of acupuncture on ischemia-reperfusion injury may vary depending on the timing of the treatment.

## 10. Regulation of related signaling pathways

Micro RNAs (MiRNAs) are endogenous small nucleotides, among which microRNA-214 (miRNA-214) plays a pivotal role in ischemia-reperfusion injury. Decreased levels of miRNA-214 can lead to an increase in calcium ion concentration potentially resulting in myocardial contraction disorders. This highlights the modulation of the endogenous cardiac protection mechanism against ischemia-reperfusion injury via the miRNA-214/Ca^2+^ pathway.^[40]^ Animal studies have indicated that stimulation of the Neiguan point can enhance miRNA-214 expression while reducing Ca^2+^ expression, leading to a decrease in the expression of related downstream proteins and reducing cell death, making it suitable for clinical application.^[41]^

Studies have shown^[42]^ that during ischemia-reperfusion injury P38 MAPK activity tends to decrease but can be activated by electroacupuncture stimulation. Additionally, P44/42 MAPK expression increases during ischemia-reperfusion injury but can be inhibited by electroacupuncture stimulation.^[43,44]^ These findings suggest that electroacupuncture stimulation of the Neiguan point can modulate the MAPK pathway, mitigate cardiomyocyte injury, and ameliorate ischemia-reperfusion injury.

In areas of the cardiac mucosa and microvessels with mast cell distribution, stimulations of ischemia-reperfusion injury can trigger the release of inflammatory factors exacerbating myocardial injury. Electroacupuncture stimulation in rats with myocardial reperfusion injury has been shown to reduce the infarct area, decrease mast cell degranulation, and exert a protective effect.^[45]^ Notably, in rats where the vagus nerve is severed, electroacupuncture at the Neiguan point does not protect cardiomyocytes. However, stimulation of the vagus nerve can inhibit myocardial mast cell degranulation and protect cardiomyocytes. This suggests that the protective effect of Neiguan point acupuncture is primarily associated with the vagus nerve-mast cell pathway.^[46]^

## 11. Conclusion

According to principles of traditional Chinese medicine, the Neiguan point is believed to protect the myocardium and plays a significant role in the treatment of myocardial ischemia-reperfusion injury. Acupuncture administered at the Neiguan point prior to the onset of histopathological changes in the myocardium has been shown to mitigate tissue damage resulting from ischemia-reperfusion. Furthermore, acupuncture at the Neiguan point before clinical thrombolytic therapy has been suggested as a means to safeguard the myocardium. Before myocardial tissue pathological changes, acupuncture at Neiguan acupoint can reduce myocardial tissue damage caused by ischemia-reperfusion. Therefore, acupuncture at Neiguan acupoint can protect myocardium before clinical thrombolytic therapy. Most of the current studies are preclinical trials. However, it is important to note that both clinical and preclinical trials on acupuncture often lack quantitative standards. The intervention time and cycle may be irregular and many research indicators are commonly used without comprehensive analysis. Therefore, there is a need for clinicians to intensify research efforts on the combination of Neiguan point acupuncture with other therapies and to expand studies on related signaling pathways. This approach will facilitate the establishment of a theoretical framework for utilizing acupuncture at the Neiguan point in treatment of ischemia-reperfusion injury.

## Acknowledgments

We would like to acknowledge the hard and dedicated work of all the staff that implemented the intervention and evaluation components of the study.

## Author contributions

**Conceptualization:** Jianen Guo, Zhibin Zhang.

**Formal analysis:** Qiuping Xin, Yuman Wang, Bingran Yang, Jiaying Sun.

**Funding acquisition:** Zhixu Gao, Yan Liu.

**Investigation:** Qiuping Xin, Zhibin Zhang, Yan Liu.

**Methodology:** Qiuping Xin, Yuman Wang, Bingran Yang.

**Writing – original draft:** Zhixu Gao, Yuman Wang, Bingran Yang, Jiaying Sun.

**Writing – review & editing:** Jianen Guo, Yuman Wang.
